# Foreign Medical and Physical Science and Literature

**Published:** 1819-03

**Authors:** 


### FOREIGN MEDICAL AND PHYSICAL SCIENCE AND LITERATURE.

### *Exposition of the Doctrine of* M. Broussais.

*(Continued from p. 174.)*

M. BROUSSAIS is, however, far from wishing to controvert the opinion that several morbid affections arise from debility. This reproach has only been thrown on him by persons who, having studied his doctrine only in a superficial manner, or being acquainted with it merely by popular report, have not fully understood his ideas. Instead of repeating, in a vague manner, that this physician can see nothing but irritation, they would have acted more reasonably in adducing instances of disease of which he had mistaken the *asthenic* character: the objection would then have been more applicable, and more beneficial to science.

Let us now take a general view of the agents which modify the state of the general system, and examine their mode of action; in order that we may develop his opinions respecting those affections which are produced by debility.

The agents that excite the animal economy, in furnishing to it nutritive materials, are not very numerous; and nearly the whole of them are received by the digestive organs. Now, they may, either by their abundance or by their qualities, irritate the membrane that lines these organs; they may produce inflammation of the blood-vessels when transmitted into them; and the parts to which they are carried are also exposed to irritation from their presence. On the other hand, when the nutritive materials are insufficient in quantity, or are to a certain degree improper for nutrition, the animal economy falls into a state of debility, which often proceeds to actual disease. Other agents, such as cold and humid air, painful moral affections, &c. frequently debilitate the system without producing irritation. The continued use of innutritious excitants, also, and increased action of organs, will produce local irritation, and induce weakness of the parts. Thus, the eye, on being exposed to a too-powerful light, becomes insensible; the genital organs, when too much exerted, are rendered lax and incapable of action, &c.

Is debility of an organ, as well as increased irritation, susceptible of being communicated to other parts? and, if so, to what extent may the propagation of it be productive of general disease? M. Broussais had not himself considered this question; and, when it was presented to him, he deferred the elucidating it to a future period. If it be true that weakness of the stomach is sometimes propagated to the rest of the body, and that want of energy in the nervous system induces a state of asthenia in other parts, it is not less certain that a communication of debility is but very rarely witnessed in practice. Indeed, the rest of the system remains uninfluenced by debility of the external parts of the body, whilst it partakes so readily of their irritation; and it appears to be affected by weakness of the internal organs only in cases where there results from it a privation of nutritive materials, or direct want of irritation; as when the functions of the digestive, respiratory, or nervous organs, are diminished.

However it may be with regard to the above question, general debility, however great, is never an obstacle to the existence of local irritation. So far from it, that increased action of the different organs appears, in some measure, to be more readily induced, in direct proportion to the greater degree of weakness of the patient. We know with what facility convalescents contract inflammation of the mucous membranes; what extreme susceptibility there is to irritation in persons debilitated by excessive hæmorrhage, &c.

When irritation has existed for a considerable length of time in any organ, the tissues analogous to it become gradually disposed to contract the same affections. This proposition is, as M. Broussais observes, one of the fundamental principles of pathology; it is a law which serves to explain how chronic inflammation of the pleura, for instance, is so often communicated to the peritoneum; how irritation of the mucous membranes of the stomach and intestines becomes connected with a similar affection of the mucous membrane of the lung; how disease of one part of the fibrous system, as gout and rheumatism, is followed by inflammation of other parts of a similar structure in succession; and, lastly, in what manner irritation of the blood-vessels or lymphatic glands of one part of the body, is so frequently communicated to the whole of their respective systems. It is this disposition to the succession of irritation which constitutes, in the opinion of M. Broussais, what have been termed the inflammatory, rheumatic, cancerous, and scrofulous, diatheses. He rejects as erroneous the admission of an altered state of the fluids, termed a depravation of them, which has so long been regarded by pathologists as the material cause of these affections; but, from his opinions on this point having been objected to, we shall develop the arguments he has brought in support of them when we treat of scrofula and other chronic morbid changes of structure.

The diseases which ensue from organic irritation may terminate in different ways, independently of their passing into a chronic form, and propagating themselves to other parts of the affected tissue:—1. The disturbance of the nervous system in general, which is the consequence of the pain of the irritated organ, may be sufficiently violent to produce death, before local inflammation may be developed. Thus, we have seen in experiments with the oxy-muriate of mercury, animals perish from the effects of pain, before the poison had induced inflammation of the stomach.—2. The violent re-action of the sanguineous and nervous systems may give rise to mortal congestions or hæmorrhage, more or less abundant.—3. On the local irritation gradually diminishing, and the symptoms dependent on it following the same course, the state of health may be re-established.—4. After a greater or less period of duration, on the irritation rapidly diminishing, its resolution is followed by sudden re-establishment of the equilibrium of the system and the appearance of the excretions previously suppressed: the organic actions and the evacuations, which signalize the last mode of termination, constitute what have been termed *crises.* Let us stop an instant to examine this subject.

Physicians have been for a long time divided respecting the determination of the exactness of the critical days of Hippocrates, and on the importance that should be attributed to crises in the practice of medicine. The doctrine of the philosopher of Cos has been considered as incorrect by a great number of judicious practitioners. According to M. Broussais, we should consider crises as the consequence of the cessation of local irritation, which permits the return of action throughout the system: it is, he says, a phenomenon similar to the return of heat in a part in a state of health, after extreme cold, violent passion, or a copious meal that has induced shivering; only this disturbance appears to be morbid in consequence of its violence in degree.

The actions observed in crises are more violent, and the evacuations which follow them more abundant, in direct proportion to the strength of the patient, the intensity of the disease, and the shortness of its duration. We should never in practice, M. Broussais observes, wait for these salutary natural effects. In general, practitioners do not rest in expectation of crises in pneumonia, pleurisy, manifest inflammation of the stomach, &c.; every one agrees that our efforts should be directed to the immediate removal of the inflammatory action. It is only, he continues, in some fevers, and in diseases the nature and seat of which are unknown, that we are recommended to wait without interference for the appearance of critical evacuations. But, he also remarks, these pretended essential fevers are really the consequence of irritation of the gastric organs, and their progress should not be inactively contemplated, any more than that of other instances of symptomatic febrile re-action.

The partisans of the doctrine of crises had not hitherto opposed any arguments, deduced from rational physiology, to the attacks of their adversaries: they relied on the testimony of the correctness of the opinions of Hippocrates, furnished by the experience of ages; and from having themselves observed that critical evacuations, happening on the indicated days, were followed by convalescence. These physicians have made of this subject, as well as of many others, a sort of sacred tradition, to which we could not refuse our assent without being guilty of medical impiety. No person, however, contests the correctness of their observations, and those of their predecessors. M. Broussais himself acknowledges their propriety; but the consequences which have been deduced from them are not always favourable to the interest of the patient. Indeed, when a disease is left to itself, in expectation of the crisis, it sometimes carries off the patient before this can happen to save him: in a great number of instances, the vital powers being exhausted by the violence of the disease, the critical actions are not manifested, or are incomplete; and very often the irritation that constitutes the disease is only dissipated to be carried to some important organ, where it may be as dangerous as in the first; which constitutes metastases not unfrequently fatal. "The conclusions that may be deduced from these reflections," continues M. B. "show that inactive expectation of crisis presents a greater number of unfavourable chances, than a vigorous mode of treatment properly directed."

Having thus disclosed to our readers a view of the doctrine of M. Broussais in its more general application, we shall proceed to the consideration of it with respect to lesions of particular organs.

Two courses here present themselves to us: we might take, with M. Broussais, a nosological system, and, passing through it, adduce his opinions respecting each of the morbid affections comprised in its different divisions; but, in proceeding thus, we should give to these articles an extent and a polemic form, which are not conformable to the plan we have proposed to adopt. The second way that is open to us consists in examining successively the diseases of the vascular and nervous systems in the different organs, and developing the opinions of the author of the *Examination* respecting them. In adopting the latter method, is it indifferent with what part we commence?—We think not. Affections of the *gastric system* being complicated with all others, those which should particularly engage the attention of physicians, and, indeed, constituting the basis of the pathology of M. Broussais, are the maladies which we shall examine in the first instance.

The beautiful discoveries of modern zoölogists have shown that the digestive canal is the most important part of animal organization, and that it is this which forms, in a manner, the characteristic of animal life. On attentively considering the functions of this part of the system, we perceive that, as the object of almost all the actions of an animal is to supply it with matter adapted for its use, so are its functions connected, in the strictest manner, with the general economy. It is, then, not surprising that the digestive viscera have the most extensive and immediate sympathies with the nervous system of *animal** life and the loco-motive organs. The most eminent physiologists long since observed, that the states of plenitude and vacuity, of health and of disease, of the stomach, exerted a powerful influence on both the moral and physical faculties of man. It is well known to every observant practitioner, that, after a too copious repast, when the stomach is overcharged,

* This term is here used in its peculiar sense, according to the doctrine of Bichât.

a sensation of weight in the head, general weakness and depression, and pains in the joints, are commonly experienced.

M. Broussais has profited by the consideration of these facts, and, passing from physiology to pathology, he has shown that the same sympathies which, in slight indispositions, announce increased excitement of the stomach, characterize the morbid state of this viscus, when they are exaggerated by a more violent degree of irritation. Another circumstance, which adds to the importance of the gastric system in the eyes of the physiologist, is, that it is to that part of the economy that the greater number of medicines are immediately applied. How can we (says M. Broussais) deposit thus, on the extremely-sensible mucous membrane of the digestive organs, substances which are intended to affect the most distant parts of the body, if we are not well acquainted with the signs and effects of their immediate influence? We shall see that, according to that philosophical enquirer, local affections in parts distinct from the gastric system, and which are sufficiently violent to induce *fever*, only produce this effect by sympathetic irritation of the heart and the mucous membrane of the stomach and small intestines: it thence results that there is no severe disease that is not complicated with this irritation, or of which the progress and treatment will not be modified by the same cause.

All organic irritations present a crowd of anomalous circumstances, which are the source of innumerable varieties in the characters of local affections and sympathetic disorders dependent on them. The extensive modifications of individual constitutions are so many causes which produce equally numerous variations, in different persons, in the character and violence of the symptoms of disease. Thus, inflammation of the tissues surrounding the articulations, in gout and rheumatism, is sometimes highly painful, at others almost indolent; in many cases it produces violent febrile re-action, in others no general emotion ensues. Under how many different aspects do irritation of the brain, lungs, and pleura, present themselves. The stomach and small intestines are, as well as all other parts of the body, susceptible of various modifications of excitation; and, by means of their extensive sympathies, disorders of those parts produce innumerable varieties of morbid affections in different individuals. But, whilst assiduous study of inflammations of other membranous parts has shown us the true nature of many of them, the inflammatory character of which had been misunderstood,—such as croup, diarrhœa, dysentery, &c.—the affections of the gastric system had hitherto been considered only in a very partial manner: the seat of many of them being unknown to the greater number of physicians; and of the generality of them (says M. Broussais) we possess only some confused ideas of the organic modifications on which they depend.

*Gastric embarrassment; gastric* and *mucous fevers;* gastritis, properly speaking; plague; *adynamic*, *ataxic*, *typhous*, and *yellow*, fevers; are nothing else, according to M. Broussais, than various species of inflammation of the mucous membranes of the stomach and small intestines, differing in their degrees of violence, as well from the peculiar constitution of the patient, as the causes which may have produced them. We cannot here trace the symptoms of each of those affections, since to effect it would lead to details which we must avoid, as well as to a repetition of circumstances generally known: we shall confine our attention to a rapid view of the characteristic phenomena of the whole, and then point out the causes of the numerous and different appearances which they present.

M. Broussais terms these maladies *gastro-enterites*, not because he believes that, in all cases, the stomach and intestines are irritated in the same degree, but because the affection which commences almost constantly in the first of these organs is quickly propagated to the second; and from the curative indications being the same, whatever may be the part most violently affected. He endeavours to point out, from the symptoms, the principal seat of irritation in the different stages of the disease: when it is violent, and has continued for a certain length of time, he has constantly observed that it has been communicated throughout the whole extent of the superior portion of the digestive canal. The large intestines are, however, ordinarily free from disorder; and, when they are implicated, the particular symptoms give notice of it to the attentive practitioner.

All the modifications of the *gastro-enteritis* of M. Broussais present the following symptoms, somewhat modified in different instances:—loss of appetite; more or less of urgent thirst; and various degrees of derangement of the digestive functions. In all of them we observe, on the centre of the tongue, a coating, which is variable in thickness, density, and colour; and, about the point and lateral parts of that organ, a redness that varies in colour from a rose-tint to the most fiery hue: appearances noticed by many authors, and to which M. Broussais particularly directs our attention, as to one of the most positive and constant signs of gastric irritation. The pulse, which is not affected in slight affections, is frequent, sharp, and contracted, in proportion to the violence of the local inflammation: it becomes weak, and falls below the natural standard, when the disease has been caused by a deleterious agent,—when the patient has been previously exhausted,—when the inflammation has attained a great degree of intensity,—and, lastly, when gangrene has taken place. The heat of the skin is increased, particularly about the abdomen and the epigastric region, and conveys a sensation of roughness to the hand that is one of the most constant effects of irritation of the mucous membrane of the digestive organs. This heat follows, in general, the modifications of the frequency of the pulse, being augmented or diminished in a relative manner: sometimes, however, the same relation does not take place; and this circumstance, which is only the result of sympathetic derangement of the nervous system, has been adduced as one of the principal evidences of the malignancy of the diseases above enumerated. The nerves, and the cerebral centre in particular, are influenced in various ways and in different degrees: there is commonly depression of spirits, morosity, and more or less violent cephalalgia; and these symptoms may extend to the most profound stupor and depression of the nervous power: at other times, on the contrary, there is an exaltation of the functions of the brain, which vary from a slight degree of wildness of manner, to the most furious delirium. Lastly, one of the most remarkable sympathies with the stomach and small intestines is evinced in the extremities: in all cases of gastro-enteritis, there exists a sensation in the muscles similar to that arising from fatigue, and often violent and almost insupportable pains in the joints. When the disease has attained a great degree of intensity, the consequent sympathetic affections increase in violence, and we then observe, conjoined with debility of the nervous system, a prostration of the voluntary powers of the muscles, with an exaltation of the action of those organs, accompanied with the most terrible convulsions.

If we examine the most frequent causes of those diseases which we should (according to M. Broussais) regard as the results of different stages and varieties of existing irritation of the mucous membrane of the stomach and small intestines, we shall find that they are such as act, either directly or indirectly, on the digestive system. Thus, repeated errors of regimen, the ingestion of acrid and irritating substances, the influence of putrid miasmata, &c. appear in the first order: amongst those of the second may be counted those miasmata which, being received into the system through the skin or by the respiratory or digestive organs, always evince their influence on the latter; excessive heat of the atmosphere, which excites the skin and, sympathetically, the stomach and small intestines, &c.

There is not one of the different stages of gastro-enteritis which may not pass to another, either more or less violent in degree. Thus, what is ordinarily called *gastric embarrassment*, as all authors have observed, is very often the precursor of *gastritis*, in the common application of this term,—gastric, mucous, and ataxic, fevers, &c. which succeed each other in the above order in the same individual. The causes that produce typhus, yellow fever, and plague, sometimes confine their influence to the determination of a slight degree of gastric irritation. Now, in the progressive increase of the symptoms which characterize the passage of the malady from the most trifling to the most violent form, it is absolutely impossible to observe any exact period at which the affection precisely changes in its nature: every thing, on the contrary, indicates that it is the same organic lesion acquiring more intensity, and producing more alarming sympathetic affections. We can see no reason which should authorize us to divide the collection of symptoms into two, three, or more sections, and to say that two or three different maladies have succeeded to each other.

Can we derive, from the effects of medicines, any knowledge which may lead us to recognize the nature of diseases? We must be very adverse to the formation of conclusions on this basis; the sensibility of different persons being so variable, that the most opposite remedies have appeared to be attended with success in similar affections. But, if we consider the effects of different modes of treatment from extensive series of observations, we may acquire some useful information, on the correctness of which we may be allowed to depend. M. Broussais has shown that antiphlogistic measures are those which most frequently succeed in the maladies of which we treat: the fortunate results he has obtained from their application are beyond any comparison in extent with the success that has ensued from contrary modes of treatment. He has frequently seen tonics and sedatives, successively applied to the digestive canal, exasperate and moderate those affections by turns, and occasion them to proceed with rapidity from the slightest to the most violent degrees, and *vice versâ.* Are not all our writings on medicine filled with observations which show the succession of gastric, adynamic, and ataxic, fevers, to gastric embarrassment, under the influence of stimulants? What do these terms signify, but that the same affection has become exasperated? If it be not so, there must necessarily be groups of symptoms, modifications of the animal economy, replacing each other: a vague and gratuitous explanation.

If the examination hitherto made of gastro-intestinal irritation has indicated what is the true nature of the affections we have enumerated, we now arrive at a source of information which will not permit us to continue in doubt respecting them. Pathological anatomy, indeed, aided by an attentive consideration of the character of the symptoms, furnishes the most positive and incontestible illustrations of the nature of those diseases. We should think, on a slight view of the subject, that, from the opportunities of exploring the bodies of those who have fallen victims to inflammation of the superior part of the digestive canal having unfortunately been too frequent, those affections should have been long since well understood: such, however, has not been the case.

On the termination of gastro-enteritis, or of the numerous maladies which represent the different degrees of that affection, we always find the consequences of inflammation of the digestive canal; but the aspect and nature of these remains of organic irritation present several varieties, the knowledge of which is of considerable importance. When the disease has been but of short duration, we sometimes find the tunics of the stomach and intestines injected with blood, and presenting throughout their whole extent, even on their external surface, a rose-coloured hue that is not natural to them, without discovering manifest inflammation of any determined portion of the mucous membrane. This shows that these organs have been the seat of irritation that has occasioned an inordinate afflux of blood to those parts, but that inflammation had not developed itself. We may compare this state to that of apoplexy, where the vessels of the brain are found distended with blood, without any effusion having taken place. It would not, however, be right to represent to ourselves the mucous membrane as little coloured with blood during life as we may find it after death: the tongue is very often of a bright red colour in the patient, but becomes pale and discolored in the dead body. It seems that irritation must have continued for a considerable length of time to render the colour of the parts permanent after death; that is to say, to combine a certain quantity of the red fluid with their tissue.

After this slight degree of lesion of the gastric viscera, we successively witness a more or less deep-red colour, or a brown or blackish hue, of the mucous membrane; the latter of which indicates the near approach to, or actual occurrence of, gangrene. Very remarkable occurrences, that are frequently noticed in the bodies of persons who have died from gastro-enteritis, are invaginations, more or less extensive, of the intestinal parieties. These foldings, which have so long been considered as a peculiar affection, under the name of *volvulus, ileus,* &c. are regarded by M. Broussais as the result of the violent and irregular contractions that agitate the small intestines. According to him, practitioners in general have, in these cases, mistaken an effect for a cause; they have considered that as an essential affection which is only a consequence of irritation of the gastric viscera.

In general, whatever may have been the disorder observed in the superior parts of the intestinal canal, the inferior portion (that is, the large intestines,) is unaffected. But, when gastro-enteritis has been accompanied with diarrhœa or dysentery, we find traces of inflammation of the latter parts also: this extent and complication of organic lesion constitutes a severe form of disease.

*(To be continued.)*

---

*General Reflections on Fistulæ, and on the Formation of an accidental Membrane in their Course; followed by some Observations collected from the Clinical Lectures of* Professor Dupuytren *on the different Species of Maladies of this kind, and on the particular Mode of Treatment adapted for them.* By M. Breschet, Prosector to the Faculty of Medicine of Paris, and first Clinical Assistant at the Hôtel Dieu.

(From the *Journal Universel des Sciences Medicales,* June 1818.)

We passed over this Memoir in our History of the Progress of Medicine, from the consideration that it comprised but little information of absolute novelty; but, on further reflection, we have judged that it may prove useful to many of our readers, as a development of some opinions advanced by Mr. Hunter, which do not appear to have been sufficiently reflected on by surgeons in general.

" The term *fistula,* in its more general acceptation, designates a *deep sinous ulcer, with callous edges, communicating with the*

*external surface or an internal cavity by means of a narrow opening; and from which a quantity of purulent matter is evacuated disproportionate to the extent of the ulcer.* As soon as matter of any kind, whether a recrementitious, excrementitious, or gazeous, fluid, from causes which it is unnecessary to consider in this place, happens to desert its natural course, the tissue in which this matter is diffused becomes the seat of inflammation. This inflammation is violent in degree in direct relation to the more or less irritating qualities of the fluid, and the excitability of the parts with which it has accidentally come into contact.

In consequence of the irritation produced by the presence or passage of this fluid or gas, which acts as a foreign matter, suppuration in the parts subject to its influence becomes established. The abscess opens sooner or later, either externally or into an internal cavity, according to varieties in the natural efforts or the artificial measures that have been employed. The purulent matter evacuated always presents some of the characters of the liquid that determined its formation, which escapes in combination with it, in a greater or less quantity. The continual passage of these fluids produces, in the surfaces of the artificial passage thus established, a permanent irritation that is sufficient to prevent the cicatrization of its parieties.

The presence of an animal fluid in any part not destined by nature for its reception, is then the cause both of the establishment and of the perpetuation of fistulous canals.

We shall not be surprised at such a result, if we reflect that milk, a fluid apparently so mild and incapable of producing irritation, induces violent inflammation, often terminating in suppuration and even gangrene, when it is injected into the interstices of the cellular membrane. Thus, a substance which, when applied to the mucous membrane of the digestive organs of an infant, only causes the degree of irritation necessary to affect its assimilation, becomes, in a part, the sensibility of which has not a due relation to its properties, an active morbific cause, in consequence of the extreme degree of irritation that it provokes.

There is no tissue nor organ of the animal economy, in the substance of which fistulæ may not be found. Thus, we have seen them traverse muscles, aponeuroses, and tendons, as well as the cellular and cutaneous structures. The viscera are not exempt from them; we frequently witness them in the parenchymatous organs, and occasionally in the brain.

It is, however, the cellular tissue in which they are most frequently observed, either in consequence of the ready passage it affords for liquids, or from the general distribution of this structure throughout the body. This tissue is, indeed, universally diffused; it is interweaved throughout every part, and enters, as an element, into the composition of every organ. In some instances, it serves as a medium of connexion between several other tissues, which by their union compose a single organ; in others, it establishes limits to the respective viscera, muscles, &c. and supplies them with particular or common envelopments. It is very abundant about the excretory canals, around which it forms cellular sheaths; and presents varieties in its particular texture according to the functions it is designed to perform. For instance, were fat to accumulate in the vicinity of the mucous canals, it might lessen or totally obstruct their passage; and therefore it is of a filamentous texture in those situations, not having that degree of laxity which admits of the accumulation of animal oil in its interstices. In consequence of such a disposition, that tissue favours the formation of excretory canals, and does not offer obstacles to their dilatation.

As soon as an animal fluid, having deserted its natural course, becomes accumulated or passes into this tissue, all the phenomena ensue that accompany the irritation which we have described. But this irritation also causes a remarkable change in the nutritive functions of that structure; the surface of it which is in contact with the effused fluid becomes converted into a membrane very analogous to the common mucous membranes. After the irritation has existed for a considerable period of time, and has attained a greater degree of intensity, it also induces a further change in the accidental membrane we have described, which possesses that character ordinarily termed *callosity.*

According to Dr. Baillie,* the celebrated Hunter long since observed, in his Surgical Lectures, "that the internal surface of fistulæ have an appearance similar to that of a secretory membrane, and which may be compared with that of the urethra.† This important observation seems to have had little influence on the minds of practitioners for a considerable period; but the great progress that has been lately made in pathological knowledge, and the frequent opportunities possessed of examining dead bodies, have verified and generalized the proposition of the English anatomist."

I shall adduce the following as one among the numerous facts which tend to substantiate the above statement:—

A young man, 20 years of age, had for some days perceived an indolent fluctuating tumor in the left groin, without discoloration of the skin, which disappeared after he had remained for some time in the horizontal posture. It was evidently a collection of

---

* See his *Morbid Anatomy.*

† "We find something analogous to this in the Treatise of Mr. Hunter *on the Blood, Inflammation, and Gun-shot Wounds;* but there is a wide difference between a mere assertion and the demonstration and complete history of the organization of a part. However, we shall transcribe the passage referred to:—

"I believe that a deep wound, such as that from a gun-shot, on proceeding to suppuration and forming a fistulous ulcer, becomes in some degree analogous to an excretory canal, having the power of producing peristaltic motions from the bottom to the external opening."

matter transmitted to it from some other part. It burst spontaneously, giving exit to a considerable quantity of fetid purulent matter. The pains which the patient had suffered about the vertebræ before the formation of the tumor in the groin, and which continued after it had opened, with the nature of the matter evacuated, clearly pointed out the primary disease to be caries of the vertebræ. The patient lived about two months after this time. The following were the appearances noticed on the examination of the body:—The lower dorsal and two upper lumbar vertebræ were in a carious state. A membranous canal, about an inch in diameter, extended from that part to the opening in the groin, the internal surface of which was of a bright red colour. Blood could be squeezed from it by pressure, the same as from a mucous membrane in a state of irritation. The surface of this canal was covered with purulent matter, which was furnished both by the parts about the carious bones and its own internal membrane that was irritated by the constant passage of the acrid purulent matter from the former source. It was easy to separate the artificial membrane which formed this surface, in the same manner as the internal membrane of the stomach may be rendered distinct, by dissection.

From the preceding observations, and other analogous considerations, it would appear that there can be no doubt respecting the nature of the adventitious membrane formed in fistulæ. If we now revert to the general theory of fistulous passages, we shall be obliged to admit that the present state of our knowledge of pathological anatomy would lead us to consider these diseases as dependant on the formation of an accidental tissue, which, by its organization, properties, and functions, has the strictest analogy with the natural mucous membranes.

Let us, then, enter into a general consideration of the seat, development, organization, properties, and functions, of this adventitious mucous structure.

1. It has been seen that fistulæ may be formed in all the different tissues of the animal economy, but that they most frequently are seated in the cellular membrane, which is that most extensively distributed throughout the body. This membrane is found thickly dispersed about the margin of the anus, in the perinæum, around the stenoid duct, &c.; which are the situations where fistulous ulcers most commonly appear.

2. The formation of the adventitious mucous membrane takes place, with more or less rapidity, in a direct ratio to the greater or less violence of the irritation of the tissue throughout which the extraneous fluid is diffused. The irritation is, however, not proportionate in extent to the apparent properties of the effused fluids; and it also varies in consequence of the different degrees of susceptibility of the parts affected.

The cellular membrane, in all cases, assumes in the first instance an ulcerated aspect, and furnishes a greater or less quantity of purulent matter. After this, it gradually evinces some peculiar character. It becomes red, in consequence of increased vascularity; its vital properties are exalted; the nutritive functions of the parts are changed; its density is augmented; and, finally, its appearance has become entirely changed. By means of these successive modifications, it assumes the state of a red villous membrane; differing not only from the cellular tissue, which is arranged in areola; and the serous membranes, which are diaphanous and constantly disposed in the form of close pouches; but also from all the other species of structure that enter into the formation of the animal economy. In proportion as it is developed, it becomes more and more similar to the mucous membranes. The pus furnished by its internal surface is succeeded by a mucous secretion, which is more abundant as the new membrane produces less of purulent matter. A period at length arrives when it ceases to form the mucous secretion, which may be readily discovered by preventing the fluids that originally induced it from passing through the canal that it forms.

This canal, by means of its internal surface, is connected with these fluids, and the mucous secreted by the adventitious membrane; on its external surface, it forms a boundary to the surrounding parts, from which it is, however, separated by a layer of cellular membrane that varies in point of thickness. Superiorly, it commences from a natural excretory orifice, or some surface presenting the conditions proper to constitute a fistulous ulcer; and, lastly, it constantly terminates inferiorly on some one of the cutaneous or mucous surfaces.

3. The organic elements of the adventitious membranes of fistulæ have the greatest analogy with those of the mucous membranes. This membrane is separated from the surrounding parts by a greater or less extent of cellular tissue, which may be termed *sub-mucous*; and it contains a portion of that structure in its composition, as may be demonstrated by maceration. Its redness discovers the presence of a large quantity of blood-vessels that terminate on its surface by exhalants, the existence of which is shown by the secretion of fluids. Since nutrition is performed in this membrane, we cannot doubt that it contains absorbent vessels. But it is only by means of new researches that we shall be able to determine, in a precise manner, the respective proportions of the different vessels that enter into its composition.

Notwithstanding these traits of analogy, the adventitious membranes of fistulæ differ from the natural mucous membranes in so remarkable a degree as to prevent the admission of their perfect identity.

In the first place, the adventitious membrane wants that cuticle that is observed on the exposed surfaces of the mucous membranes of the lungs, digestive organs, &c. Besides which, it does not contain those glandular bodies, termed *mucous follicles*, that are dispersed throughout the primitive mucous membranes, and secrete a viscous fluid destined to lubricate their surfaces.

These are not the only differences of organization noticed by the attentive observer between the accidental and natural mucous canals. There is a constant tendency to the obliteration of the former, as soon as the passage of the fluid through them is intercepted; whilst in the latter, under similar circumstances, such an occurrence is never, or very rarely, observed. For example, compare an old fistula, of whatever kind, provided it is still susceptible of being cured, with what is observed in cases of preternatural anus, and these different results will be very evident. We shall sooner or later succeed in effacing a fistulous canal, after having diverted from it the passage of the fluids by which it was produced, (the effecting of which, we may observe in passing, is the basis of the treatment of those diseases;) but, in the preternatural anus, on the contrary, although the whole of the fæcal matter may pass through the accidental opening, the inferior part of the intestinal canal does not become obliterated, but continues to furnish a greater or less quantity of mucous matter.

This difference with respect to the facility of obliteration of canals formed by accident, and the almost constant impossibility of effecting it in those which are lined with natural mucous membranes, shows what little expectation should be formed of the permanence of canals formed in these tissues; and how preferable are natural to artificial views in the treatment of many diseases of those parts: that is to say, those which re-establish the primitive course of fluids, to those which effect new and artificial modes of transmitting them. It is in consequence of this that passages formed through the prostate gland, by means of conical sounds, only remain as long as they are kept open by the presence of the instrument. It is from this, also, that practitioners have ceased to employ the methods of Woodhouse, Hunter, Monro, &c. for the treatment of fistula lacrymalis, and adopt that by which they endeavour to restore the natural passage of the tears. However, nothing is more common than to witness the return of that disease, when it has been supposed to have been cured by a long-continued use of tubes, bougies, &c.; but this arises from the disorder, in these cases, depending less on a contraction of the soft parts, or excretory ducts, than on that of the hard parts surrounding those passages. What proves this is, that the insertion of metallic canulæ, which offer more resistance to the bony parieties than the mucous membranes, is sufficient for a prompt and radical cure of a malady too often rebellious to all the efforts of the most able surgeons, before they had recourse to canulæ that were permitted to remain in the parts, the advantages of which were long since pointed out by M. Dupuytren; and by means of these he has cured many hundreds of cases, for which all the ordinary methods had been employed in vain.

4. The vital properties and functions of the adventitious membrane formed in fistulous passages, much resemble those of the tissue to which we have compared it with respect to its organization. This membrane possesses various degrees of sensibility. Sometimes the introduction of a probe or sound into a fistula is productive of severe pain; whilst, at others, the presence of those instruments is more readily borne. We have observed that this membrane secretes a mucous matter, which at first is mingled with pus, but afterwards flows away perfectly pure; and finally ceases to be secreted, if we divert from it the fluid or other matters that had induced it: were it not for this, we could not hope to effect the obliteration of the fistulous canal.

After having given a rapid, though exact, history of the internal membrane of fistulæ, it remains for us to ascertain, more correctly than we have hitherto done, the nature of the *callosities*, the production of which we have attributed to the permanence of the local irritation, kept up either by means of the fluid that constantly passes over the membrane, or by various external causes,—such as certain topical applications, exercise on horseback in cases of fistulæ about the anus and urinary passages, &c.

These *callosities* have a whitish appearance, and seem to be the result of congestion of colourless fluids in the membrane and subjacent cellular tissue. Formerly they were considered to be of a schirrous, or even cancerous, nature; and these erroneous notions gave rise to injurious measures in the surgical treatment of this disease. Experience has shown that rest, emollient applications, and appropriate dressing carefully applied, will, in the greater number of instances, effect their removal. According to the present received ideas respecting cancerous affections, and the treatment that should be opposed to them, it will be readily perceived that they differ in every respect from the callosities of fistulæ.

By a judicious use of the measures I shall presently point out, we may almost always spare the patient an operation, which, although generally exempt from danger, is productive nevertheless of severe pain, and frequently gives rise to very unpleasant consequences. I allude to the treatment of fistula by extirpation, a cruel operation no longer practised, or at least only adopted in places remote from the centres of acquisition in knowledge, and by persons governed by prejudices, or ignorant of the progress of the art of surgery.

A simple operation is now generally substituted in the place of extirpation, which is executed in various ways, subordinate to accidental circumstances and the particular inclinations of the surgeon. These consist, in all cases, in re-establishing or dilating the natural passages, in dividing or compressing the fistulous canal throughout its whole length, so as to give a free issue to the pus, and to oppose the passage of liquids, aëriform matters, or fluids, in the accidental canal; the bottom of which should be the first part that should become united. They have also another object, that of removing external extraneous substances, and favouring the separation and entire removal of those formed in the diseased tissues.

Fistulous passages differ considerably, according to the laxity, organization, and nature, of the cellular tissue in which they are developed. They vary also in the disposition and direction which they assume. Thus, they do not always extend in a right line; and often form numerous sinuses, terminating in more extensive cavities. The accumulation of fluids in the latter gives a complicated character to the disease, and renders the treatment of it tedious in consequence of the difficulty experienced in the discovery of these cavities, and the means of arriving at them. These complications depend on the same causes as the principal disease. The retained fluids, also, according to their nature, are productive of inflammation, and the numerous and various consequences that the practice of surgery daily offers to our observation.

We may, I believe, conclude from what has been stated—

1. That fistulæ are accidental canals, kept up by the continual passage of excrementitious substances, purulent matter, the liquids coming from secretory organs, or gazeous fluids, which produce irritation of their surfaces and prevent their adhesion.

2. That one of the extremities of fistulous passages constantly receives or produces the cause of the irritation that perpetuates their existence.

3. That it is to the knowledge of this cause that the practitioner should direct his attention, if he would employ a rational and efficacious mode of treatment.

4. That there is always the formation of a tissue of a particular nature in fistulous passages, more analogous to the mucous membranes than any other species of structure.

5. That, in some cases, this tissue is the only existing organic alteration; and this is what is commonly observed in simple fistulæ.

6. That, in many other cases, there exists at the same time a degeneration of the cellular structure and adjacent parts; a degeneration which we should be careful not to consider as the cause of the fistula, nor to confound with schirrous, cancerous, or carcinomatous, affections, from which it essentially differs.

7. That the removal of the parts thus degenerated will not remedy the cause of the fistula; it only destroys one of its effects that would generally disappear spontaneously after the cause of it had ceased to exist.

8. That the indications for the treatment of this malady are to prevent the formation of pus, or at least the flow of it through the fistulous passage; to re-establish the course of the fluids or secretions through their natural channels; and to prevent the escape of air by the opening that communicates with one of the extremities of the fistula.

9. Lastly, that, after the causes are removed, we may obliterate the fistulous passage, by dividing it throughout its whole extent; by the use of compression; or by exciting, by means of caustics or irritating injections, such an inflammation of its parietes as may induce their mutual adhesion."

### *An Essay on the Effects of Temperature on the Human System;* by Franklin Bache, M.D. of Philadelphia.

We insert this Essay without any particular comments; for, since Dr. Bache adopts the opinion that caloric is an absolute substance, and we are disposed to consider the phenomenon of heat merely as the result of a modification of *atomic motion*, our endeavours to refute such of his propositions as we may not be disposed to admit, would necessarily assume so much of a polemical form, as to recal to the minds of some of our readers the disputations in *alma mater*, and would be productive of but little utility or gratification to the enquirer. We must, however, observe, in general terms, that this Essay contains many observations and judicious reflections that merit the most careful examination and profound meditation.

"All agents which exert an operation on the human system have been arranged under the two great classes of stimulants and sedatives: there is reason, however, to believe that this arrangement is the result rather of a partial and superficial view of the subject, than one resting upon strict and logical investigation. In the mode of operation of positive agents, there is indeed a difference which may constitute the basis of a very natural division of them; but this division throws no one of them into the class of sedatives, in the abstract meaning of that term.

This difference in the manner of operation of positive agents, just alluded to, consists in the various degrees of velocity with which they produce their effects. When their operation is comparatively quick, they give rise to what is called excitement, and are stimulants in the common acceptation of the term: when it is comparatively slow, they likewise give rise to excitement, produced however in so slow and imperceptible manner as not to be obvious, but to be inferred by a concomitant expenditure of the excitability: when a stimulant has an operation of the latter kind, it is in common language denominated a sedative.

The term sedative is here used to express the action of a stimulant slowly operating. When applied to digitalis, it has this sense; but it has another acceptation, when used to signify the negative operation of privative qualities: thus it is said that darkness and cold are sedatives.

In this last sense, its acceptation is exclusively relative: it has relation to a state of greater stimulation; and in that view it implies a diminution of excitement. This will appear evident, when it is considered that we are acquainted with no absolute privative qualities; for small degrees of heat or light are called sedative, only because their operation is privative in relation to that of the usual and grateful quantities of these fluids.

If these views be correct, it would appear that, although the use of the term sedative in this last sense may be convenient on many occasions, yet, in a strict scientific investigation, it should be discarded: it can be to no purpose to say, that the diminution of any stimulant, acting on the human system, has the effect of lessening excitement, because that such must be the effect is evident; and, merely because language has supplied names to express certain relative privations, there is no shadow of reason why their negative effects should be embodied in a scientific arrangement, and suffered to assume the character of positive operations.

Thus much was thought necessary to be said as introductory to the consideration of the effects of heat, whether in excess or defect, upon the human body; for, the principal point of controversy relative to heat being on the manner of its operation during abstraction, it was deemed proper, before discussing it, to give an outline of the doctrine of stimulants and sedatives which would be assumed in the reasonings of this paper.

The matter of heat is undoubtedly stimulant; any accession of it to the human system shows this effect. The abstraction of the same fluid produces effects also: as the result of the operation of which agency shall we consider them, whether of the stimulant or sedative.

The opinion is held by many physicians, that the abstraction of heat, inasmuch as it produces very obvious effects, must be stimulant; and by some these effects are supposed to indicate a positive nature in cold, and to give probability to the opinion of the existence of frigorific particles. On the contrary, others denied that any stimulant effect arose from cold.

It must be acknowledged that a good deal may be said in favour of each of these opinions; for, upon an abstract logical consideration of the question, the mind is certainly compelled to conclude that the abstraction of heat could not be stimulant; and yet the arguments used to prove that it is, have the appearance of great plausibility.

It will appear, however, upon reflection, that this difference of opinion among physicians has arisen from their omitting to analyze the phenomena which occur upon the abstraction of heat from the human system. This neglect has prevented them from seeing the subject in that two-fold point of view which is essential to its proper comprehension; for, with a view to its effects upon the human system, the matter of heat may be considered, 1st, with reference to its relative excess or defect; and, 2dly, with reference to its velocity during accession or abstraction: considered with reference to its excess or defect merely, it is either stimulant or sedative; but, with reference to its velocity, always stimulant.

The human system in the healthy state is supplied, from an internal source, with a quantity of the caloric of temperature, superabundant when compared with that which is usual in the atmosphere: it is, therefore, in all common cases, undergoing, like heated bodies, the process of refrigeration. At a temperate heat of the atmosphere, one at which the refrigeration of the human body goes on neither so fast as to decrease its usual quantity of floating heat of temperature, nor so slow as to allow it to accumulate, no uneasy feeling is produced; for here the velocity of the caloric, in passing off, is such as to stimulate irritability, but not sensibility, in consequence of the influence of habit; and the quantity of floating caloric of temperature being the same at every moment, it affords a constant and equable stimulus.

If the heat of the external air be a good deal less than temperate, the refrigeration of the body goes on faster than in ordinary times; consequently, the caloric, in leaving the surface, has a greater velocity than usual, and, by impinging upon the solids and fluids in passing out, stimulates both sensibility and irritability, and its impression upon the former produces the sensation of cold. As soon as the heat of the exterior of the body is diminished to such a degree as that by diminishing the rate of refrigeration, the loss of heat is not greater than the supply, in equal times, the sensation of cold ceases; since the cause of the sensation, namely, the increased velocity of the caloric in leaving the body, is removed. Hence it is that a person, upon going out of a warm room into the cold air in winter, feels at first the sensation of cold upon his face, but after some time that part becomes of a natural temperature to his sensations. This does not prove, however, that it is really of the ordinary animal heat; for, when touched by the hand of a person who has been in a warm room, it will give indications of a minute state as to temperature.

By the same mode of investigation, the effects of heat upon the body, when the temperature of the surrounding air is suddenly increased above temperate, may be traced in all their stages. Here, then, the state of refrigeration is first lessened; the heat of the body is next accumulated; whereupon the state of refrigeration is proportionably increased, until at length, by the recurrence of the original difference in temperature between the air and the body, the former rate of refrigeration is again established, and further accumulation prevented. The body now possesses an accession of caloric; yet, after the usual state of refrigeration is restored, there is reason to believe that the sensation of heat is considerably diminished.

In thus tracing the phenomena which attends the process of refrigeration in the human body, it is not meant to deny to it the power of regulating the production of its heat according to the state of its wants: on the contrary, it is believed that the human system has this power, but it is presumed that it cannot be exerted fully on a sudden exigency.

The subject may be further illustrated by the following examples:—

If the hand be placed in hot water, it is stimulated, as well because it receives an accession of heat, as that it receives that fluid moving with considerable velocity: if it be placed in water of a medium temperature, which is gradually increased, it is stimulated by the accession of heat only, its velocity during accession not having any appreciable effect. On the contrary, if the hand be plunged in very cold water, the operation, considered as one of the abstraction of heat merely, is certainly sedative; but, considered with reference to the velocity of the caloric in leaving the hand (which by the supposition will be considerable), it must be deemed a stimulant. Again, if the hand be plunged into water of its own temperature, but which is gradually cooled down to a low one, then the operation, considered as an abstraction of heat, has the effect of a sedative; and, considered with reference to the velocity of the caloric during abstraction, it has no perceptible effect of any kind.

In addition to those stated in the foregoing theory, one more mode, in which heat may stimulate during abstraction, will be suggested for consideration. May not this fluid, under such circumstances, stimulate indirectly by allowing a fuller energy of the attraction of cohesion between the particles of the solids and fluids of the body to take place? The elasticity of the matter of heat certainly affords the principal antagonist force to this attraction; and any circumstance, with respect to the motion of caloric, which should have the consequential operation of bringing the particles composing the human body into more energetic contact, must have, in that view, a stimulant effect.

The view here taken of this subject gains much evidence in its favour by considering the injurious effects produced by caloric, whether in excess or defect: it will be found that these depend almost exclusively on its velocity. The impression made by the contact of frozen mercury is said to resemble very much that from the contact of red-hot iron: now the effects produced in these cases cannot depend upon the amount of caloric lost or received, because that must be inconsiderable; they must, therefore, result from the disorganizing operation of the matter of heat traversing the solids and fluids with great velocity.

It will be found, upon examination, that almost every case which is adduced in proof of the stimulant operation of cold, is such a one as, by its conditions, supposes not a mere abstraction of heat, but an abstraction of that fluid with considerable velocity; while, on the other hand, cases of the gradual and almost imperceptible abstraction of heat are adduced in proof of the sedative operation of cold. Does it not hence appear probable that the true explanation of the great difference in the effects produced by abstracting heat in various cases is to be sought in the different velocities of the departing caloric under different circumstances?

There can be no well-founded reason for believing that the stimulating effects produced by the motion of caloric are modified by the direction in which such motion is established: on the contrary, it is very probable that the stimulus given to sensation by what is called heat and cold depends so entirely upon the velocity of caloric, that nearly, if not precisely, the same sensation would be produced by the same velocity of that fluid, whether established in the centripetal or centrifugal direction, with respect to the human body; and, taking for granted that the process of refrigeration is regulated by the same laws at all temperatures, if any part of the human body could be cooled down to 16 degrees below zero without injury, such part would be as completely scalded by a sudden plunge into water of a temperature equal to the animal heat, as it would be, under ordinary circumstances, by immersion into that fluid at the boiling point.

The peculiar view which is here taken of the effects of heat upon the body attaches great importance to its velocity, and points to the necessity of an exact investigation of the laws which regulate the heating and cooling of bodies; it also supplies a more rational explanation of the manner in which animals endure exposure to high temperatures without injury; and, as this question is very particularly connected with the subject of this paper, it may be proper to examine the experiments of Sir Charles Blagden, made to investigate this point. All his experiments went to prove that the human body could bear the contact of air, heated to 30 or 40 degrees above the boiling point of water, without much inconvenience; and the exposure to this great heat never produced a variation of more than a few degrees in the animal temperature. The singular results of the experiments were explained by Sir Charles Blagden, partly from the slow manner in which air communicates its heat, thereby allowing sufficient time for the human body to destroy it, and partly upon the process of evaporation: but his view of the subject will be better understood in his own words.

In the first paper,* Sir Charles Blagden, after enumerating the experiments, has these words:—"These experiments, therefore, prove in the clearest manner that the body has the power of destroying heat. To speak justly on this subject, we must call it a power of destroying a certain degree of heat, communicated with a certain quickness. Therefore, in estimating the heat which we are capable of resisting, it is necessary to take into consideration, not only what degrees of heat would be communicated to our bodies, if they possessed no resisting power, by the heated body, before the equilibrium of heat was effected, but also what time that heat would take in passing from the heated body into our bodies." He goes on further to say:—

"In consequence of this compound limitation of our resisting power, we bear very different degrees of heat in different mediums. The same person who felt no inconvenience from air heated to 211 degrees, could not bear quicksilver at 120 degrees, and could just bear spirit of wine at 130 degrees: that is, quicksilver, heated to 120 degrees, furnished more heat for the living powers to destroy than spirit heated to 130 degrees, or air to 211 degrees. And we had in the heated room, where our experiments were made, a striking, though familiar, instance of the same: all the pieces of metal there, even our watch-chains, felt so hot that we could scarcely bear to touch them for a moment; whilst the air from which the metal had derived its heat was only unpleasant."

In the concluding sentences of this quotation, the principle upon

---

* See *Philosophical Transactions*, vol. lxv. part i. p. 119.

which the human body is capable of enduring a high temperature is made manifest. Let the circumstances of the body, when exposed as above described, be examined, and it will appear that the caloric of temperature of the skin, not being in a balanced state with respect to the heat of the air, was constantly receiving additions from that of the latter; but it was also in a minus state with relation to that of the watch-chains. Why, then, did the contact of the air, or of the metallic bodies, produce such different effects? Most assuredly, because the caloric of temperature issued from the latter bodies with considerable velocity in its tendency to come to a state of rest; whereas, this same kind of caloric leaves air comparatively slow, when influenced by the same tendency.

It would, therefore, appear that these experiments go very much to prove, in confirmation in part of the theory advocated, that accessions of heat merely, unattended by great velocity of that fluid, have comparatively little effect in stimulating the human system.

But, in reviewing the experiments performed in the heated rooms, it will be recollected that the animal temperature varied very little: how is this to be accounted for? The body could bear slow accessions of the heat of temperature from other bodies, without increasing its own heat of temperature: the heat, therefore, must have changed its state upon entering the human system; it must have assumed the form of latent caloric,—that state of this fluid in which it remains at rest, undisturbed by the plus or minus state of the caloric of temperature in surrounding bodies.

It is this capacity which the human body seems to possess under peculiar circumstances of rendering caloric latent, which Sir Charles Blagden calls a power of destroying heat. Dr. Dobson, however, suggests the explanation on the doctrine of latent heat.

If it be correct to suppose that the caloric became latent in these experiments, it must nevertheless be conceded that the body has a limit to its power of rendering it thus latent; and, although the human system may, by an exertion of its preservative power, increase its own capacity for heat, yet this increased capacity must be constantly approaching to a state of saturation: and hence, if the persons who were the subjects of the experiments, had remained sufficiently long in the heated rooms, there is fair reason to presume that, after a certain period, the animal temperature would commence to be gradually increased, until it became balanced with that of the surrounding heated medium.

After making these remarks, as giving the most probable explanation of the results of these experiments, it must not be concealed that some of the phenomena observed appear to militate against it. The conversion of the heat of temperature into latent heat has here been considered as depending upon an exertion of the vital power, not as taking place at the surface, and consequent upon the chemical laws of evaporation; yet the slow heating of certain kinds of dead matter, under equal circumstances of exposure to heated air, rather favours the opinion that possibly the maintaining the equal tenor of the animal temperature in such cases may depend upon the same principle. What these appearances were will be seen from the following extracts from the experiments:—

Sir Charles Blagden mentions his having placed an earthen vessel containing pure water in one of the heated rooms, the temperature being perhaps 20 or 30 degrees above the boiling point: he goes on to say, "In one hour and a half the pure water was heated to 140 degrees of the thermometer." A little farther on he remarks, "The pure water never came near the boiling point, but continued *stationary* above an hour at a much lower degree." But afterwards he tells us, that, by dropping a small quantity of oil into it, the water at length came to boil very briskly.

Hence it would appear that pure water has a power, under circumstances of exposure to air heated above the boiling point, of resisting an increase of its temperature beyond a certain degree; for, in the above extract, it is stated that the temperature of the water experimented upon became stationary much below the boiling point. This depended, as Sir Charles Blagden remarks, upon the process of evaporation, which must be supposed to have taken place when the water was at the stationary point of temperature, at just such a rate as to be sufficient to render latent any accession of heat as soon as received. The truth of this explanation is rendered almost certain, since, upon the addition of oil (which acts by preventing evaporation), the water came at length to boil briskly.

Why, then, it will be asked, if evaporation from water should be sufficient to prevent an increase of temperature beyond a certain point in that fluid exposed to hot air, may not the evaporation from the human body, under similar exposure, prevent the increase of the animal temperature?—more especially, since it is probable that the same balance of received heat on the one hand, and absorbed heat by evaporation on the other, which takes place with respect to water when exposed to hot air at a comparatively high temperature, may occur, with respect to the human body, at the common animal heat.

Sir Charles Blagden admits, that, upon exposure to hot and comparatively dry air, the evaporation must prove a powerful assistant in keeping the body cool; but he cannot believe that it is alone sufficient to keep it at a uniform temperature, for this reason, which is here given in his own words:—"Evaporation can act only in the gross way, and by no means in such nice proportion to the momentary exigencies of the animal as would be requisite for an exact preservation of its temperature." But it would appear that, if the explanation, on the principle of evaporation, was liable to no other difficulty than the one stated as above quoted, it could be satisfactorily made out; for, if the dryness of the air should remain the same, then, by a kind of physical necessity, the evaporation would be directly proportional to the heat of that medium at any moment, and would vary as to its amount upon every alteration, however small, of the heating cause.

But the strong objection to the explanation exclusively upon the principle of evaporation, is derived from the experiments detailed in Sir Charles Blagden's first paper; for here he informs us that the body, immersed in hot air loaded with vapour, so far from suffering any process of evaporation from its surface, acted as a condenser of the moisture held by the surrounding air, to such a degree that streams of water poured down its surface on all sides; and under these circumstances, also, the body preserved its animal temperature; but it must be noticed that the temperature of the heated air was not very considerable. Now it was satisfactorily ascertained that the aqueous fluid which appeared was not sweat, but condensed vapour, since a similar condensation took place upon the surface of a Florence flask filled with cold water, upon being brought into the heated room.

These experiments were, indeed, attended with curious results; but it is doubtful whether they afford any satisfactory explanation of the manner in which the human system can maintain an uniform animal heat, under circumstances of exposure to comparatively high temperatures. The nature of that power still remains enveloped in difficulty, and will perhaps continue so, until investigated with great diligence by means of other experiments in heated rooms, which should be varied in every possible manner, in order to elicit a consistent explanation.

*Recapitulation.*—1. All agents operative upon the human system are necessarily stimulants.

2. Stimulants differ in the promptitude with which they act. Some act quickly, others comparatively slow. They may be arranged in gradation, according to the promptitude of their action, from the quickest to the slowest in operation.

3. By one sense of the term sedative, a stimulant slowly operating is expressed.

4. By another acceptation of this term, the operation of a stimulant relatively defective is conveyed. The term is objected to in this sense; since an agent, which was a sedative at one time, might be a stimulant at another.

5. With a view to its effects on the human system, heat may be considered, 1st, with reference to its relative excess or defect; 2d, with reference to velocity during accession or abstraction. Considered with reference to the former, it is either stimulant or sedative; but with reference to the latter, always stimulant.

6. Quere? May not the abstraction of heat be indirectly stimulant, by allowing a fuller energy of the attraction of cohesion to take place?

7. The stimulating effects of caloric, as far as they depend on motion, are not modified by the direction, whether centripetal or centrifugal, in which such motion is established with respect to the human body.

8. The view here taken attaches particular importance to the velocity of caloric, in judging of its effects upon the human body: this points to the necessity of investigating carefully the laws which regulate the heating and cooling of bodies.

9. It would appear that the human body is capable of enduring the contact of hot air without raising its own temperature; on account of the slow conducting power of air, on account of evaporation, and of the power which the body seems to possess of increasing its capacity for heat. If it has the last-mentioned power, it can exert it to a certain extent only.

10. Water, exposed to heated air, resists an increase of its temperature beyond a certain point, by the evaporation which takes place from its surface.

11. But the maintenance of the equal tenor of animal temperature cannot be explained exclusively on this principle; because the human body exerts the same resistance to an increase of its temperature, when, so far from any evaporation going on from its surface, it acts as a condenser of the vapour held by a surrounding heated medium.—(*American Medical Recorder*, vol. i.)

### *Case of Tubercular Leprosy;* by M. Alibert.

M. Dupuis, a native of the north of France, had resided eleven years at Marseilles, and passed three campaigns in the army of Italy, when he went, in 1801, to the Isle of France; where he remained nine years, enjoying good health. He had left that island several months, and was on the way to France, when, with the joy he felt in the expectation of soon seeing again his native country, he felt a dread that he should arrive there in a state of sickness. Within twenty-four hours after he had experienced the latter emotion, his body became covered with large patches of a reddish-violet colour, which were tender to the touch, but were not accompanied with derangement of the general health. On arriving home he consulted a physician, who merely directed the use of some bitter infusion and the warm-bath.

This treatment was continued for fifteen months without benefit; when some other practitioner ordered sulphurous baths, and the frequent use of purgatives. Under the influence of these means, the blotches in part disappeared, and of those that remained a change of colour was observed. Some months afterwards, tubercles, of the size of a nut, appeared on the thighs and along the spine, containing puriform matter. Another physician was then consulted, who considered the disease to be syphilitic, and directed mercurials; which were administered for a year without being productive of benefit. The patient then went to Rochefort, to see some persons who had resided in India, and were said to be well versed in the knowledge of such diseases. They recognized it as leprosy, and advised the use of sulphur and the baths of the water of Bagnères, both internally and externally. This advice was followed for two months; when the patient returned home nearly in the same state as that in which he had left it.

After this, the legs became swelled, the skin hard and covered with scales, and large tubercles appeared on the face: the voice of the patient became altered, his sight weakened, and his breath fetid. After having been two months in the hospital at Rochefort, he went to Paris. The feet and legs were then much tumified, and the skin covering those parts hard and wrinkled; it was also covered with elevations of a greyish colour, which gave to the extremities somewhat the appearance of those of the elephant. There were numerous violet-coloured blotches on the thighs, and several large pustules, covered with a thick crust confining a white, adhesive, and fetid matter. The trunk of the body was free from disease; but the face had an hideous aspect: the loss of the hair of the eye-brows, the eye-lashes, and the beard; the deep wrinkles on the forehead; the hard tubercles; the yellow crusts covering the checks, particularly on the right side; the largeness of the nose, and tumefaction of the lips,—gave to it an expression that was hardly human.

His sight was considerably weakened; odours made but a slight impression on the pituitary membrane of the nostrils, which was in a state of ulceration; the hearing was difficult; the voice hoarse, and speech hardly to be understood; and the sense of touch only remained about the extremities of the fingers, and on the palms of the hands; the sense of taste remained unimpaired, but there were two large tubercles on the middle of the tongue, which arose from the cellular tissue beneath the mucous membrane. There was considerable stiffness in the articulations of the instep, which rendered walking extremely slow and difficult. The trunk of the body alone was covered with a copious sweat during the night; there was a frequent evacuation of the urine; the pulse full, but very slow; and the sleep, though long continued, interrupted by terrific dreams.

Sulphurous baths and fumigations, and the use of sulphur and arsenic internally, judiciously exhibited, have produced so much amelioration in this disease, as to lead to an expectation that a cure of it will at length be effected.*

### *Description of* Professor Dupuytren's *Operation for Fistula Lacrymalis.*

The extraordinary and peculiar success with which the eminent surgeon above mentioned has for many years treated fistula lacrymalis, induces us to intrude on the attention of our readers (many of whom, we presume, are unacquainted with it,) a brief description of his mode of operating for the cure of that disease.

An incision is first made in the anterior part of the lacrymal sac, through which a golden canula, appropriate in its shape and dimension to the course of the lacrymal duct and the age of the patient,†

---

* *Journal complémentaire du Dict. des Sciences Med.* tome ii.

† A description of the canula could not be conveyed with sufficient accuracy, without a graphical representation of it. That instrument may be obtained from Lesueur, surgical instrument maker, Rue des Mathurins-Sorbonne, at Paris.

is introduced into the lacrymal duct, and, by means of a round-pointed stilette (having a handle two inches in length, making an obtuse angle of about 120 degrees with the other part, which is about twenty lines in length, and of a conical shape,) it is carried below the artificial opening, and reaches the level of the nasal fossa. The wound in the lacrymal sac is then covered with adhesive plaster, and usually cicatrizes in a day or two; when the disease is cured. In a few instances, however, the canula produces a degree of inflammation of the lacrymal duct and sac, which is productive of a little inconvenience; but hardly ever such as to prevent the final success of the operation. In a very few cases, also, the presence of the canula seems to produce retroverted action of the duct, and the instrument is forced upwards, and causes inflammation of the lacrymal sac: this accident is remedied by restoring the canula to its former situation, and keeping it there by pressure, if necessary. The canula (it is obvious, we presume, from the above description,) is suffered to remain in the lacrymal duct; and this appears to be the only mode of treating fistula lacrymalis, hitherto employed, that is permanently efficacious in the greater proportion of the cases of that disease.

---

